# Inhibition of myeloid-derived suppressive cell function with all-trans retinoic acid enhanced anti-PD-L1 efficacy in cervical cancer

**DOI:** 10.1038/s41598-022-13855-1

**Published:** 2022-06-10

**Authors:** Yun Liang, Wenshan Wang, Xiaojun Zhu, Minghua Yu, Caiyun Zhou

**Affiliations:** 1 Department of Surgical Pathology, The Women’s Hospital, School of Medicine, Zhejiang University, Hangzhou, 310006 Zhejiang China; 2 Department of Gynecology, the Women’s Hospital, School of Medicine, Zhejiang University, Hangzhou, 310006 Zhejiang China

**Keywords:** Cancer, Immunology, Medical research, Oncology

## Abstract

PD-1/PD-L1 inhibitor treatments are relatively inefficacious in advanced cervical cancer patients. The presence of myeloid-derived suppressor cells (MDSCs) in the tumor microenvironment may be one significant barrier to efficacy. It has been shown that all-trans retinoic acid (ATRA) can differentiate MDSCs into mature myeloid cells. However, whether ATRA suppression of MDSCs function could enhance PD-L1 blockade-mediated tumor immunotherapy remains unknown. Here, the frequency of tumor-infiltrating MDSCs in cervical cancer patients was measured. ATRA was used to target MDSCs both in vitro and in tumor-bearing mice. The impact of ATRA on the human cell line HeLa was also investigated. The frequency of MDSCs and T cells was determined by flow cytometry. The expression of immunosuppressive genes was measured with quantitative real time-PCR and infiltration of immune cells was assessed by immunohistochemical examination. We found that tumor-infiltrating PD-L1^+^ MDSCs were more prevalent in cervical cancer patients. Blockade of PD-L1 expression in MDSCs with anti-PD-L1 antibody cannot relieve the suppressive activity of MDSCs induced by HeLa cells, while ATRA efficiently abrogated the suppressive activity of MDSCs. Furthermore, ATRA had no effect on PD-L1 expression in HeLa cells in vitro. In in vivo treatment, ATRA decreased MDSCs accumulation and increased the frequency of CD8^+^ T cells in BALB/C mice with U14 cervical tumors. Importantly, a combination treatment of ATRA and anti-PD-L1 antibody further delayed U14 tumor growth and increased the proportion of CD62L^−^CD8^+^ T cells, CD62L^−^CD4^+^ T cells, CD107a^+^CD8^+^ T cells as well as IFN-γ and TNF-α levels in tumors. Our results provide a rationale for the use of ATRA to suppress MDSCs and enhance anti-PD-L1 cancer immunotherapy in cervical cancer.

## Introduction

Cervical cancer (CC) is one of the most common gynecologic cancers worldwide^1^. Although chemoradiation therapy (CRT) significantly improves survival for locally advanced cervical cancer, patients with advanced or recurrent cervical cancer have a poor prognosis even when receiving CRT^2^. With the recent success in solid tumor treatment with PD-1/PD-L1 axis blockade, immunotherapy has become a promising approach. With regard to cervical cancer, the FDA has granted approval for the use of pembrolizumab in PD-L1^+^ CC since June 2018^3^. In the limited clinical trials, PD-L1 expression in cervical cancer was high (37.8–80%), but the overall response rate to PD-1/PD-L1 inhibitors was relatively low (10–17%)^4–6^. This may be due to the immunosuppressive tumor microenvironment mediated partly by myeloid-derived suppressor cells (MDSCs)^7^. MDSCs utilize several mechanisms to suppress T-cell function, including a high level of arginase activity, iNOS, as well as reactive oxygen species (ROS) production. All-trans retinoic acid (ATRA) is a derivative of vitamin A, which can induce immature myeloid cells to differentiate into mature blood cells. Clinically, ATRA is currently used to induce differentiation of leukemic blasts into mature myeloid cells in acute promyelocytic leukemia patients^8^. Moreover, ATRA is a potentially valuable compound for cancer immunotherapy. ATRA has been used to enhance the effect of cancer vaccines^9^, antiangiogenic therapy^10^ and CAR-T(Chimeric Antigen Receptor T-Cell) therapy^11^. In this study, we investigated for the first time whether ATRA could improve PD-L1 treatment for cervical cancer in vitro and in a mouse model.

## Materials and methods

### Patient samples and cell lines

Tumor specimens were collected from 19 cervical cancer patients undergoing surgical treatments. For controls, normal cervical tissue was collected from 10 healthy age-matched volunteers. The clinical details for the patients are provided in Supplemental Table 1. All patients had not received neo-adjuvant treatment before surgery. Informed consent was obtained from all individuals before sampling and the study was approved by the ethical committee of the Woman’s Hospital School of Medicine Zhejiang University (IRB-20200338-R). All research was performed in accordance with the relevant guidelines and regulations. Human HPV-positive cervical cancer cell lines (SiHa, HeLa) and HPV-negative cervical cancer cells (C33A) were maintained in our laboratory and cultured in RPMI 1640 containing 10% FBS (Hyclone) and 1% penicillin/streptomycin (Santa Cruz) at 37 °C with 5% CO_2_. A mouse cervical cancer (U14) cell line was purchased from the Institute of Materia Medica (Chinese Academy of Medical Sciences, Beijing, China). Cells were cultured in Dulbecco modified Eagle medium (DMEM) supplemented with 10% fetal bovine serum.

### Flow cytometry analysis

Tumors were minced into small (1–2 mm^3^) pieces, and digested using a Tumor Dissociation Kit (Miltenyi Biotec) according to the manufacturer’s instructions. The resulting supernatant was filtered through a 200-mesh sieve and washed twice with ice-cold PBS^12^. healthy donors were incubated for 5 min with Fc-block (BD Biosciences). The phenotype of MDSCs in patients was determined by a combination of surface markers including HLADR-PerCP-Cy5.5 (Clone L243, BD Biosciences), CD33-FITC (Clone P67.6, BD Biosciences), CD11b-APC (Clone CBRM1/5, BD Biosciences), and CD45-BV421 (Clone HI30, BD Biosciences). Afterward, PD-L1 membrane expression was evaluated using PD-L1 (CD274)-PE (Clone 29E.2A3, BD Biosciences).

For the mouse model, single-cell suspensions were stained with either a myeloid panel of antibodies comprising CD45-FITC (Clone 30-F11, BD Biosciences), CD11b-APC-Cy7 (Clone M1/70, BD Biosciences), Gr-1-PE (Clone RB6-8C5, BD Biosciences), and PD-L1-APC (Clone 10F.9G2, BD Biosciences) or a lymphoid panel of antibodies comprising CD45-FITC (Clone 30-F11, BD Biosciences), CD4-PerCP/Cy5.5 (Clone RM4-5, BD Biosciences), CD8-PE (Clone 53–6.7, BD Biosciences), CD62L-APC (Clone MEL-14, BD Biosciences), and CD107a-APC (Clone 1D4B, BD Biosciences). Flow cytometry was performed with a BD FACS Canto II flow cytometer. Data were analyzed using FlowJo software (TreeStar, Inc, Ashland, OR).

### Induction of human MDSCs in vitro and magnetic bead isolation

HeLa cells were seeded in culture plates to achieve 80% confluence. PBMCs were isolated from healthy blood donor by Ficoll-Paque (GE Healthcare, Chicago, IL, USA) density centrifugation. PBMCs were then cultured in RPMI 1640 medium containing 10% fetal calf serum with 50%(v/v) of HeLa cell conditioned medium with or without 2 μM of ATRA (Sigma Aldrich). After 5 days, all cells were collected from the tumor-PBMC co-cultures. Adherent cells were removed using a non-protease cell detachment solution^13^. Myeloid cells were then isolated from the co-cultures using anti-CD33 magnetic microbeads and LS column separation (Miltenyi Biotec) as per the manufacturer’s instructions. Expression of the MDSC markers HLA-DR, CD33, CD11b, and CD14 was examined by flow cytometry. The CD3^+^ T cells were prepared from PBMCs in healthy donors using a Human Pan T Cell Isolation Kit (Miltenyi Biotec). The purity of the cells after sorting was > 90%.

### RNA isolation and real-time quantitative RT-PCR

To determine the expression difference, total RNA from generated CD33^+^ MDSCs or HeLa cell lines was extracted using the TRIzol reagent (Invitrogen, USA) according to the manufacturer’s instructions. Quantitative RT-PCR for Arg-1, iNOS, NOX2 and PD-L1 was performed using Maxima SYBR Green qPCR MasterMix (Fermentas). The primers were shown in Supplemental Table 2. Ct (threshold cycle) values was determined with the Applied Biosystems Step One Plus Real-Time PCR System. Data were analyzed by the ΔΔCT method^14^.

### T cell proliferation assays

Carboxyfluorescein succinimidy ester (CFSE)-based proliferation assays were performed to test the T cell proliferation. CD3^+^ T cells (100,000/well) isolated from HD were incubated with CFSE and stimulated with anti-CD3 and CD28 (both 1 μg/mL). T cells were co-cultured with sorted MDSCs at the ratio of 1:0.25, 1:0.5, 1:1 in RPMI-1640 complete medium. After stimulation for 3 days, at 37 °C, 5% CO_2_, proliferation of CD8^+^ T cells was gated by measuring CFSE intensity by flow cytometry^10^. T cells without anti-CD3 and anti-CD28 antibody stimulation were used as a negative control. For the indicated studies, MDSCs were co-cultured with T cells in the presence of ATRA or anti-PD-L1 antibody.

### Cell viability assay and cell apoptosis assay

HeLa cells were seeded in a 96-well plate at 5 × 10^3^ cells/well and incubated for 48 h with ATRA at different concentrations (1, 2, 5, 10 and 20 μM). Cell viability was assessed with a Cell Counting Kit-8 (CCK-8)^15^. Cell apoptosis was assessed with an Annexin V/PI apoptosis detection kit and BD FACS Canto II flow cytometer was used to measure fluorescence^16^.

### In vivo animal models and treatments

Female BALB/c mice (6–8 weeks old) were obtained from the Experimental Animal Center of Zhejiang University. All mouse-related experiments were approved by the Experimental Animal Welfare Committee of Zhejiang University (ZJU20200119). All methods are reported in accordance with ARRIVE guidelines (https://arriveguidelines.org) for the reporting of animal experiments and all methods were carried out in accordance with the relevant guidelines and regulations. Mouse cervical cancer U14 cells were prepared as mentioned above and a total of 1 × 10^6^ U14 cells were subcutaneously injected into the abdominal cavity of the mice for a week. The ascites was taken out of the mice and diluted with sterile normal saline to 2 × 10^7^ cells/mL. Then, 0.2 mL of carcinoma cell suspension was subcutaneously injected into the right axilla to establish the U14 carcinoma model^17^.

Six days after tumor cell inoculation (when tumor size reached 150–200 mm^3^), tumor mice were injected with ATRA (Sigma Aldrich) at 7.5 mg/kg daily around the tumor or with anti-PD-L1 mAb (clone: 10F.9G2, Bio X cell) at 10 mg/kg on days 6, 9 and 12 around the tumor. All mice were randomly divided into four groups (8 mice per group) as follows: model control group; anti-PD-L1 Ab group (injection of anti-PD-L1 Ab); ATRA group (injection of ATRA); combination group (injection of anti-PD-L1 Ab + ATRA). Tumor size was recorded every three days from the day of inoculation to day 21, when mice were sacrificed.

### Cytokine detection

Levels of murine IFN-γ and TNF-α were detected on tumor supernatants collected and analyzed were quantified by ELISA (eBioscience) in accordance with the manufacturer's instructions.

### Immunohistochemical examination

Tumor samples isolated from tumor-bearing mice were fixed in 10% formalin for 24 h prior to processing for immunohistochemical analysis. Immunohistochemical staining was performed using the 2-step Envision method according to the manufacturer’s instructions (Abcam, Cambridge, UK). The sections were incubated with antibodies against CD4, CD8 and CD11b (polyclonal; abcam; 1:100). The number of lymphocytes were expressed as cells/mm^2^.

### Statistical analysis

For the analysis of parametric and nonparametric data, we used Student's t test (two groups) and ANOVA with Tukey's post hoc test (more than two groups). All tests were performed with GraphPad Prism version 6.0 (Graphpad Software, CA, USA). P values < 0.05 were considered statistically significant.

## Results

### Tumor-infiltrating MDSCs were expanded in advanced cervical cancer patients

**Figure 1:**
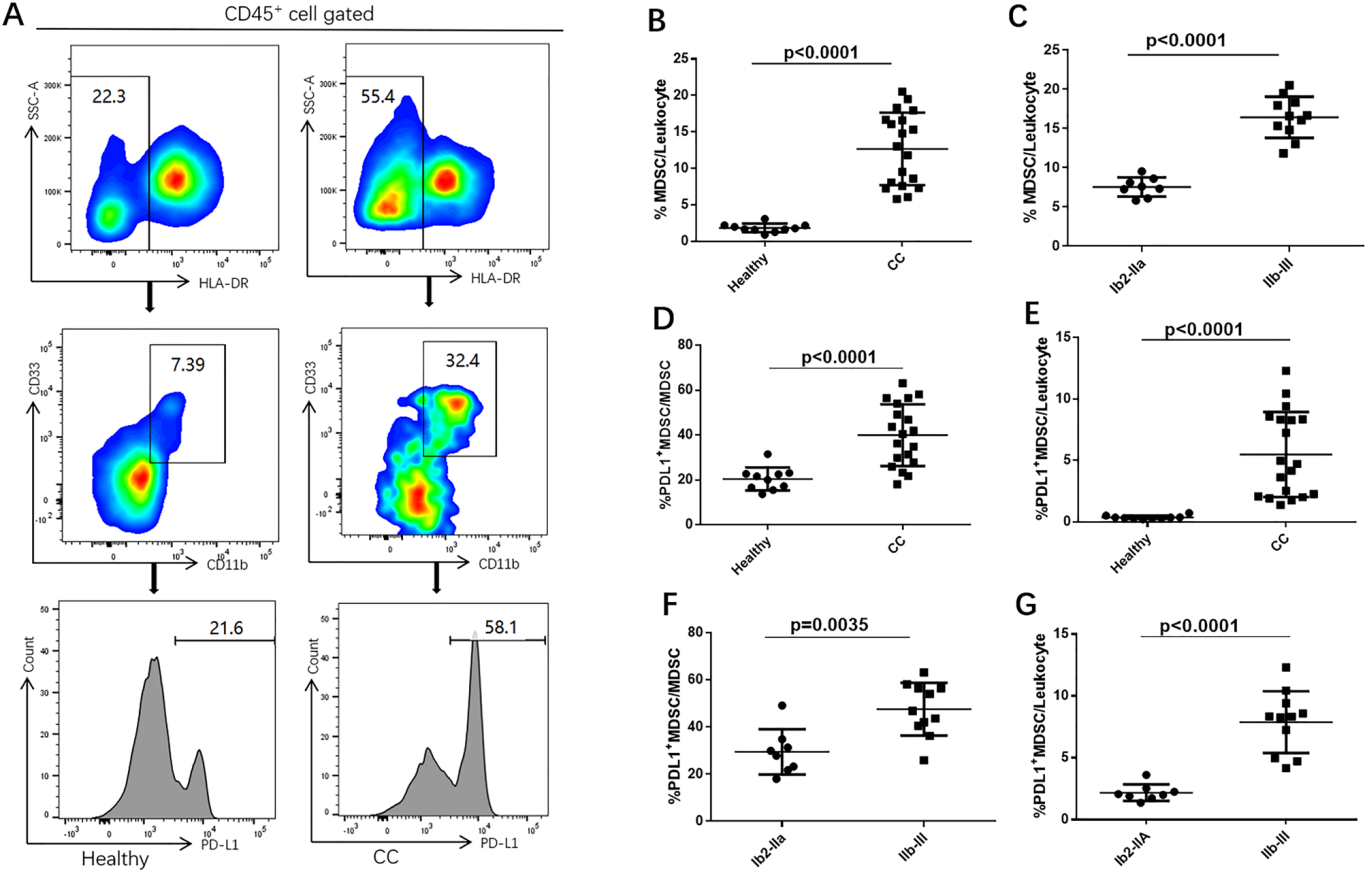
Tumor-infiltrating PD-L1^+^ MDSCs increased in cervical cancer patients. (**A**) Representative flow cytometry from a normal healthy donor (left) and a CC patient (right). (**B**) Percentages of MDSCs in CC patients were significantly higher than in healthy donors. (**C**) Percentages of MDSCs in stage IIb-III patients were significantly higher than in stage Ib2-IIa patients. (**D**) PD-L1^+^ cells in total MDSCs were significantly higher in CC patients compared to healthy donors. (**E**) Percentages of PD-L1^+^ MDSCs in CC patients were significantly higher than in healthy donors. (**F**) PD-L1^+^ cells in total MDSCs were significantly higher in stage IIb-III patients compared to those in stage Ib2-IIa patients. (**G**) Percentages of PD-L1^+^ MDSCs in stage IIb-III patients were significantly higher than in stage Ib2-IIa patients. 2 repetitions with similar results. Data are shown as mean ± SD.

To determine whether MDSCs played a role in the progression of cervical cancer, we sought the frequency of tumor-infiltrating MDSCs defined as HLADR^−/low^ CD33^+^ CD11b^+^ population (Fig. 1A). The frequency of MDSCs was calculated as a percentage of total leukocytes in single cell suspensions. A significantly higher level of MDSCs was observed in cervical cancer patients compared with controls (median 13.02% vs. 1.76%, p < 0.0001) (Fig. 1B). MDSCs levels were also higher in stage IIb-III than in stage Ib2-IIa (median 16.6% vs. 7.456%, p < 0.0001) (Fig. 1C). All these results indicated that MDSCs were linked to the progression of cervical cancer.

The percentage of PD-L1^+^ MDSCs was also investigated. PD-L1^+^ MDSCs levels (in terms of both total MDSCs and leukocytes) were significantly higher in patients than in healthy donors (median 40.5% vs 21.25%, p < 0.0001; median 4.715% vs 0.337%, p < 0.0001) (Fig. 1D,E). For patients, the PD-L1^+^ MDSCs level in total MDSCs as well as in leukocytes was also higher in stage IIb-III than in stage Ib2-IIa (median 46.8% vs 28.85%, p = 0.0035; median 8.315% vs 2.034%, p < 0.0001) (Fig. 1F,G).

### ATRA promoted the maturation of MDSCs

**Figure 2:**
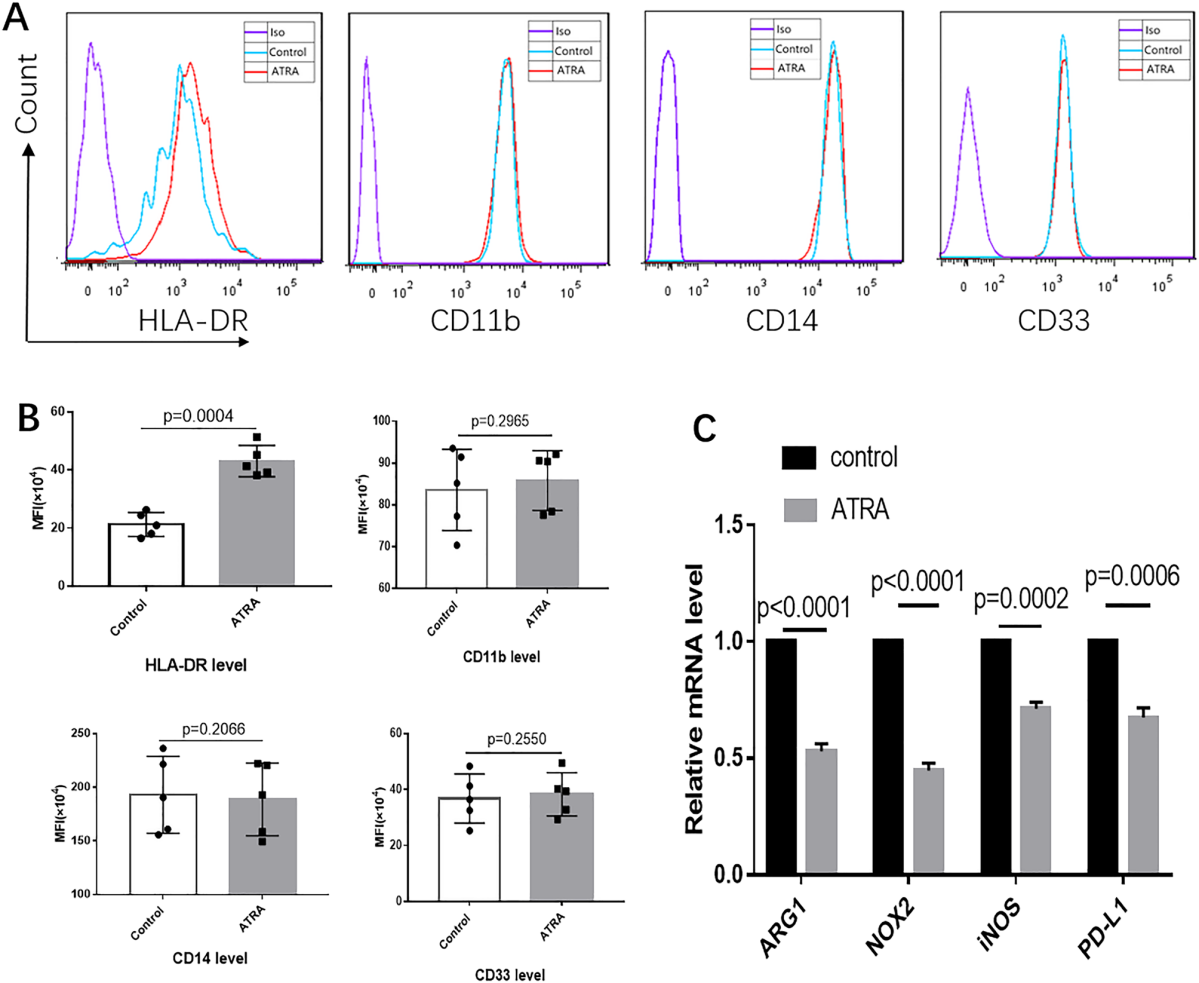
ATRA induced differentiation of MDSCs and decreased immunosuppressive capacity in human MDSCs. (**A**) Representative flow cytometric histograms for expression of HLA-DR, CD11b, CD14 and CD33 on HeLa induced MDSCs in the presence of 2uM ATRA or control compared to the appropriate isotype. (**B**) Comparison of mean fluorescence intensity (MFI) of HLA-DR, CD11b, CD14 and CD33 expression in MDSCs in the presence and absence of ATRA. The MFI level of HLA-DR increased after ATRA treatment, indicating a reduction of HLA-DR^-^CD33^+^CD11b^+^ MDSCs. (**C**) The relative mRNA expression of Arg-1, NOX2, iNOS and PD-L1 decreased in the presence of ATRA. Data are representative from 5 separate experiments. Data are shown as mean ± SD.

Studies have demonstrated that human MDSCs can be induced from peripheral blood mononuclear cells when co-cultured with a diverse set of human tumor cell lines^13^. To investigate the effect of ATRA treatment on MDSCs in vitro, we co-cultured isolated human PBMCs with HeLa cell lines for one week in the presence or absence of 2 μM ATRA. We observed that ATRA promoted differentiation of MDSCs with increased expression of HLA-DR, which indicated a more differentiated state, while expression of the other MDSC markers (CD11b, CD33 or CD14) did not change (Fig. 2A,B). We further evaluated expression of immunosuppressive genes in induced human MDSCs by real-time PCR and found that ATRA significantly reduced expression of the immunosuppressive genes Arg1, iNOS, NOX2 and PDL1 transcript levels in sorted CD33^+^ myeloid cells (Fig. 2C).

### Blocking PD-L1 expression in MDSC did not relieve suppressive activity of MDSCs in vitro

**Figure 3:**
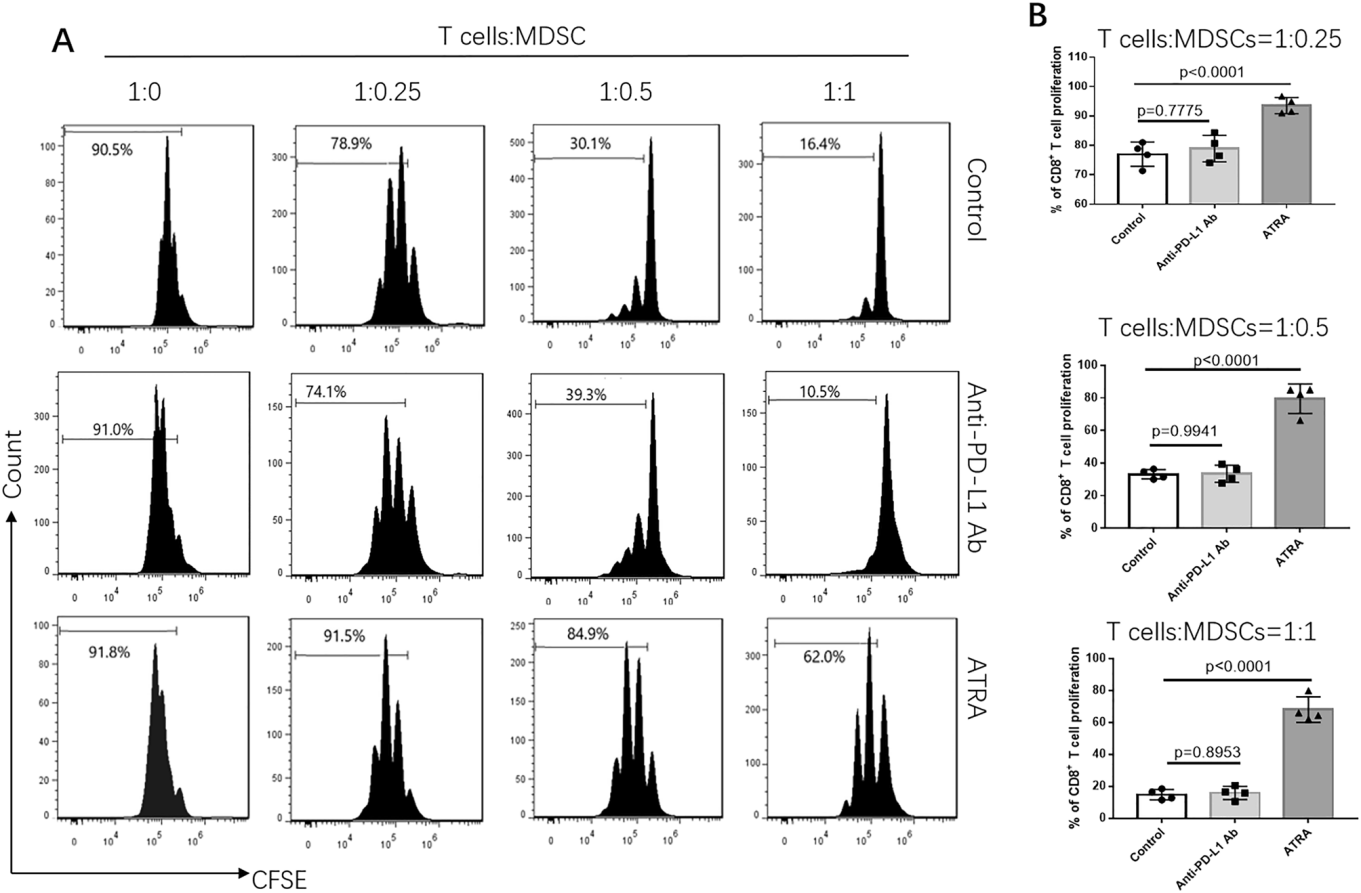
The role of anti-PD-L1 Ab or ATRA to relieve the suppression of T-cell proliferation by MDSCs. (**A**) Representative flow cytometric histograms from a T cell proliferation suppression assay. (**B**) Bar charts showing that MDSCs suppressed proliferation of CD8^+^ T cells in a dose-dependent manner (T: MDSC cell = 1:0.25, 1:0.5, 1:1). In the presence of anti-PD-L1 antibody (50 μg/mL), the decrease of MDSC-mediated suppression was limited. ATRA (2 μM) significantly reversed MDSCs-mediated suppression under different suppressive conditions. Data are representative of 4 separate experiments. Data are shown as mean ± SD.

To determine whether PD-L1 expression in MDSCs was the contributing factor to their immune-suppressive activity, we co-cultured T cells with induced human CD33^+^ myeloid cells at ratios of 1:0.25, 1:0.5, 1:1 ratio in vitro. We found that MDSCs suppressed the proliferation of CD8^+^ T cells in a dose-dependent manner. Then, we added anti-PD-L1 neutralization mAb (50 μg/mL) to the coculture system to remove PD-L1 function. No significant change in MDSCs-mediated suppression was seen in the presence or absence of anti-PD-L1 neutralization mAb (Fig. 3A,B). These observations indicated that PD-L1 activity was a minor contributor to suppressive function in HeLa-cell-induced MDSCs.

### ATRA efficiently abated suppressive activity of MDSCs in vitro

MDSCs suppressed proliferation of T cells. We theorized that if ATRA-induced depletion of MDSCs was functionally important, then equal numbers of CD33^+^ myeloid cells should be more suppressive than equal numbers of CD33^+^ myeloid cells pretreated with ARTA in vitro. We performed T-cell suppression assays, with different T cells: MDSCs ratios at 1:0.25, 1:0.5, 1:1, in the presence or absence of ATRA (2 μM). ATRA significantly reversed MDSCs-mediated suppression under different suppressive conditions (Fig. 3A,B).

### ATRA had no effect on PD-L1 expression in HeLa cells in vitro

PD-L1 expression levels in tumor cell were closely associated with the efficiency of PD-L1 antibody treatment. Therefore, we explored whether application of ATRA could influence PD-L1 expression in cervical cancer cell lines. HeLa has the highest PD-L1 expression level among cervical cancer cell lines (Supplementary Fig. 1A). We treated HeLa cells with ATRA and found that there was no change in PD-L1 expression levels in HeLa cells when the cells were incubated with ATRA at 2uM for 48 h (Supplementary Fig. 1B). On the other hand, the addition of 2 μM ATRA to cultured HeLa cells had little effect on cell viability as measured by a CCK-8 viability assay (IC50 = 8.57uM) (Supplementary Fig. 1C) or by cell apoptosis (Supplementary Fig. 2). As a result, it was clear that ATRA did not have a direct effect on HeLa cell.

### ATRA treatment reversed immune suppression by increasing CD8^+^ T cells in vivo

**Figure 4:**
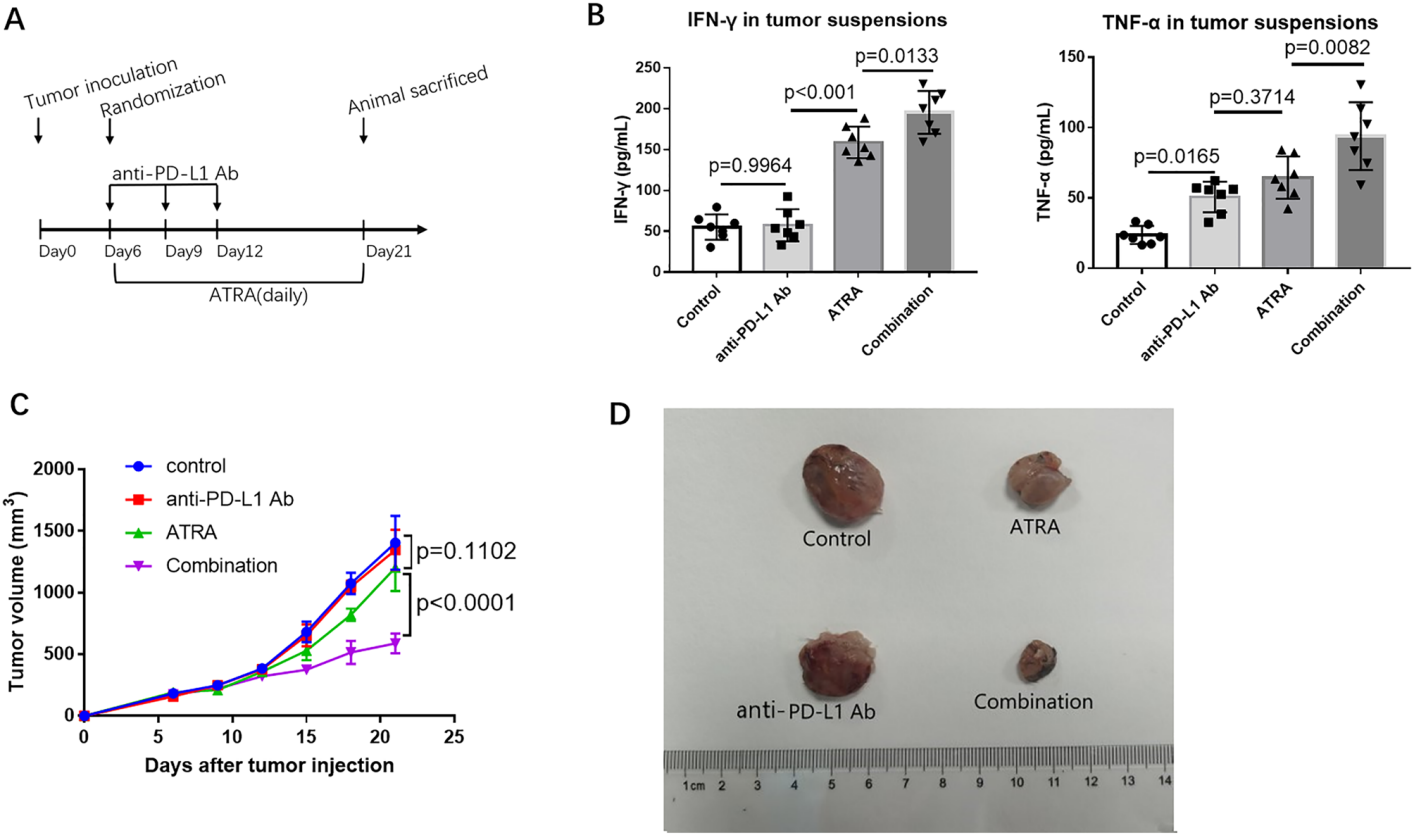
ATRA enhanced the anti-tumor activity of PD-L1 blockade in vivo. (**A**) Experimental design for BALB/c mice, injected with 0.2 mL of U14 cervical cancer cell suspension. From day 6, the mice were injected with ATRA daily, anti-PD-L1 antibody on day 6, 9 and 12, or a combination of both treatments. The mice were sacrificed on day 21. (**B**) Levels of supernatant IFN-γ and TNF-α in different treatment groups were quantified with ELISA (8 mice/group, 2 repetitions with similar results). (**C**) U14 tumor growth curve of BALB/c mice (8 mice/group) with different treatment. Tumor volume was measured and recorded every 3 days after treatment. (**D**) Representative tumor images show the changes in volume between groups on Day 21. Data are shown as mean ± SD.

**Figure 5:**
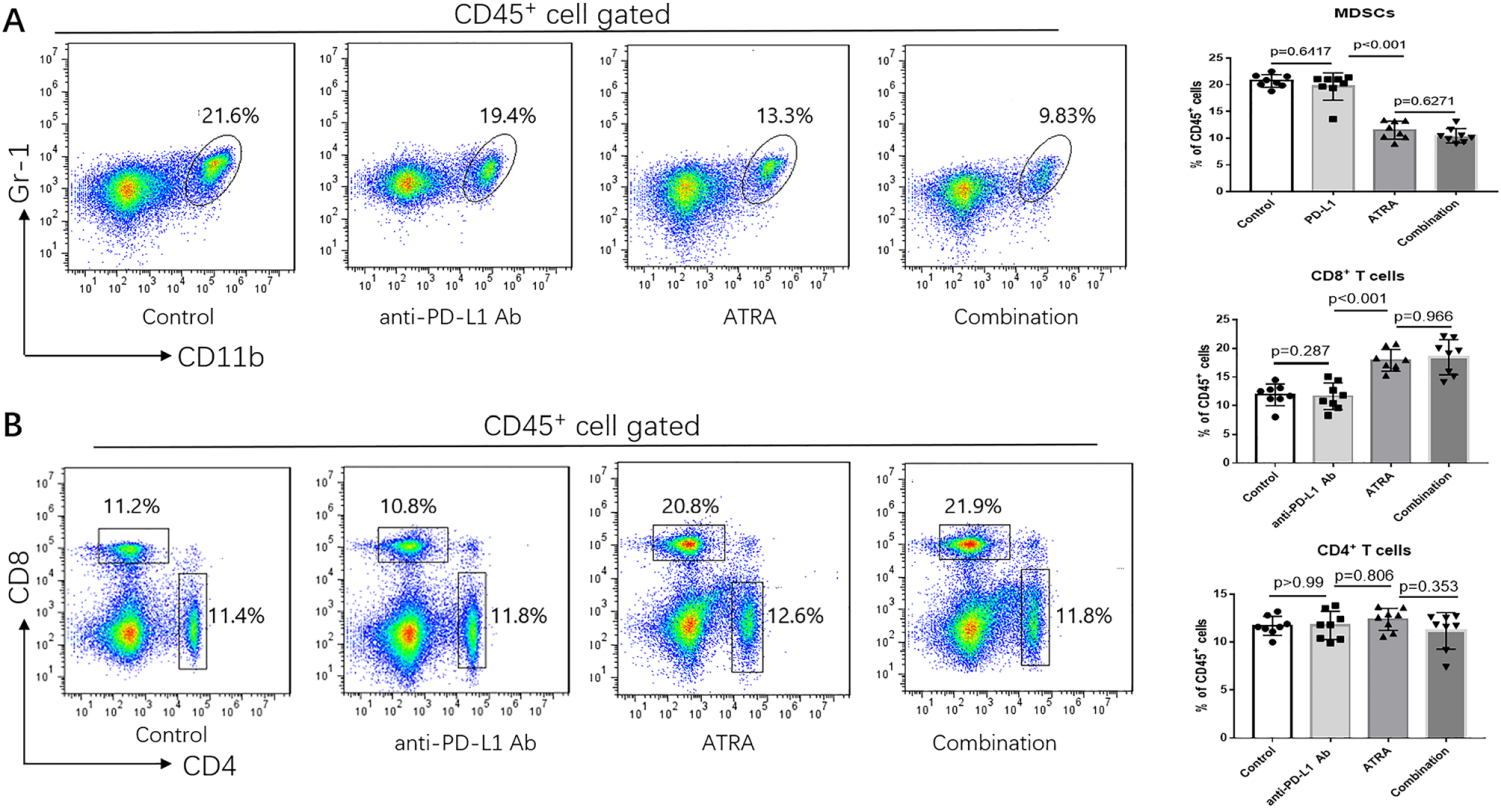
ATRA in vivo partially reversed MDSCs suppression and enhanced CD8^+^ T cell infiltration in tumor-bearing mice. At 21 days after inoculation, tumor tissues were harvested, and tumor-infiltrating CD45^+^ immune cells were examined. Representative flow cytometry dot plots of MDSCs (**A**), CD4^+^ T cells and CD8^+^ T cells (**B**) in different treatment groups(left)and the comparison of the MDSCs, CD4^+^ T cells and CD8^+^ T cells proportions in different groups (right) are shown. Data are shown as mean ± SD (8 mice/group, 2 repetitions with similar results).

**Figure 6:**
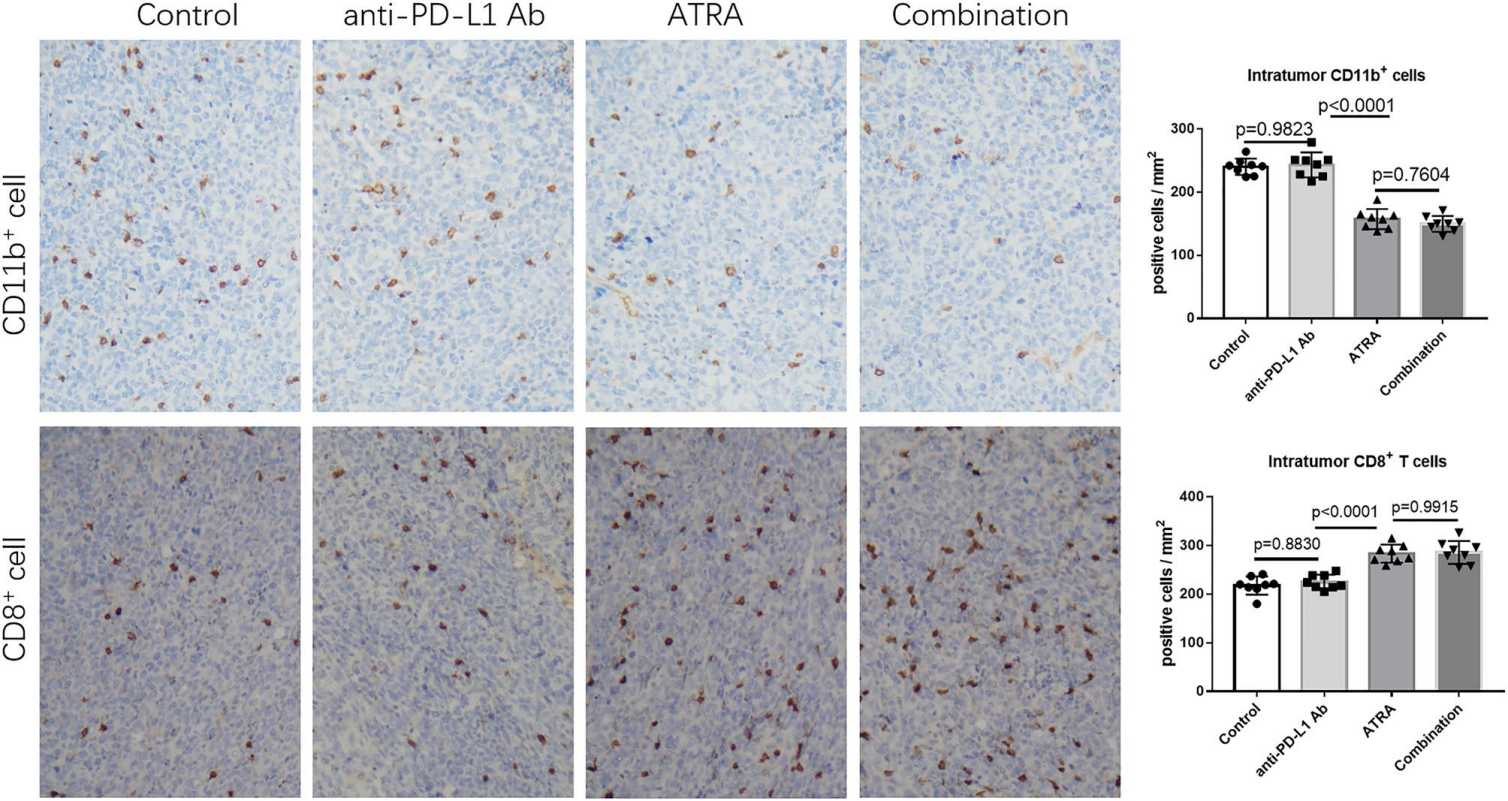
Change in CD11b^+^ and CD8^+^ cells in tumor beds after treatment. Representative immunohistochemical images of CD11b^+^ and CD8^+^ cells in the tumor beds from different groups (left) and the comparison of CD11b^+^ cells and CD8^+^ T cells numbers in different groups (right). Data are shown as mean ± SD (8 mice/group).

In order to investigate the hypothesis that ATRA could increase the efficacy of PD-1/PD-L1 antibody, we first assessed its effect on tumor growth and correlated immune factors in BALB/c mice. Based on previous studies, a dose of 7.5 mg/kg was administered in our study^10^ (Fig. 4A). ATRA treatment slightly reduced tumor growth while was no significant (Fig. 4C,D). When analyzing tumor single-cell suspensions with FACS, we found decreased accumulation of MDSCs (Fig. 5A), enhanced infiltration of CD8^+^ T cells (Fig. 5B) after ATRA treatment. No change was found in the expression of CD4^+^ T cells (Fig. 5B). The IFN-γ level and TNF-α level in the supernatant of tumor homogenates also increased, as shown by ELISA (Fig. 4B). Furthermore, using an immunohistochemical staining approach, we found that the number of CD8^+^ T cells in the tumor bed increased along with the decreased CD11b^+^ myeloid cells in the ATRA group (Fig. 6). There was no markable difference in the infiltration of CD4^+^ T cells after ATRA treatment (Supplementary Fig. 3). All of these results demonstrated that ATRA treatment altered the immunosuppressive tumor microenvironment in U14 tumor bearing mice. Interestingly, the PD-L1 expression level in tumor single-cell suspensions increased slightly after ATRA treatment (Supplementary Fig. 4).

### A combination of ATRA and anti-PD-L1 mAb suppressed tumor growth

**Figure 7:**
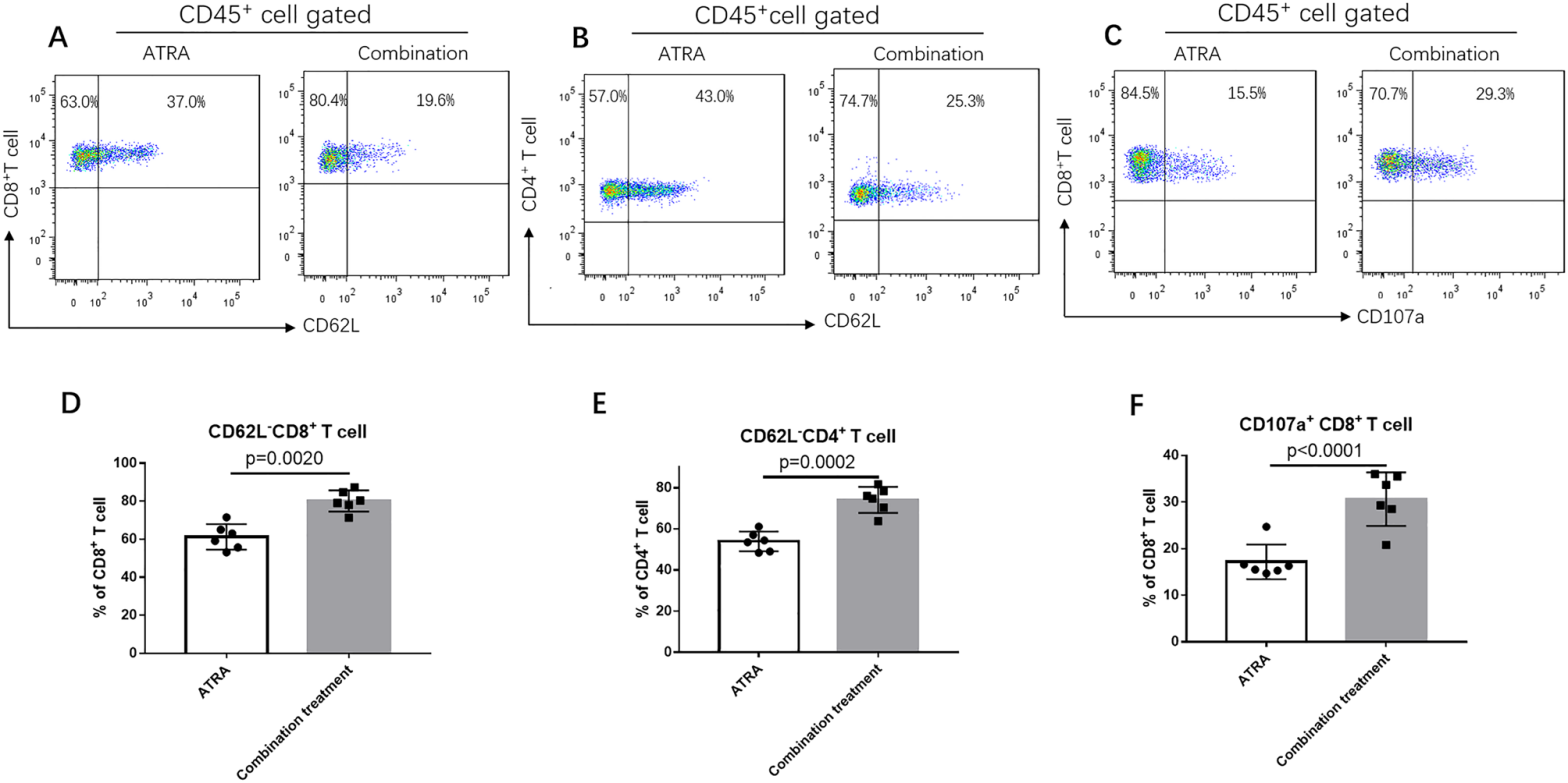
The proportion of T cells with effector functions increased in vivo after combination treatment of ATRA and anti-PD-L1 mAb. Representative flow cytometry dot plots show CD62L^−^CD8^+^ T cells (**A**), CD62L^-^ CD4^+^ T cells (**B**) and CD107a^+^ CD8^+^ T cells (**C**) in ATRA group and combination treatment group. The comparison of CD62L^−^CD8^+^ T cells (**D**), CD62L^−^CD4^+^ T cells (**E**), and CD107a^+^CD8^+^ T cells (**F**) proportions in two groups are shown. Data are shown as mean ± SD (6 mice/group, 2 repetitions with similar results).

We found that a combination of ATRA and PD-L1 antibody was superior to monotherapy with either one in reducing U14 tumor growth (Fig. 4C). However, infiltrating immune populations through FACS or immunohistochemical examination showed that the level of enhanced CD8^+^ T cells and decreased CD11b^+^ cells in the combination treatment group was similar to that of the ATRA group (Figs. 5, 6). We further explored the proportion of T cells with effector functions. Results showed that in combination treatment group, the proportion of CD62L^−^CD4^+^ T cells, CD62L^−^CD8^+^ T cells increased (Fig. 7A,B,D,E). The proportion of CD107a^+^CD8^+^ T cells also increased (Fig. 7C,F). Along with T cells gaining effector functions, the cytokine production of both IFN-γ and TNF-α significantly increased (Fig. 4B).

## Discussion

Multiple clinical trials of immune checkpoint inhibitors have strongly suggested that the tumor immune microenvironment plays a significant role in tumor progression^7,18^. In our research, the accumulation of circulating MDSCs in cervical cancer patients was associated with disease progression, which supported an important role for MDSCs in immune suppression. We also found increased PD-L1^+^ MDSCs expression in cervical cancer patients. This phenomenon was similar to that in a previous report, which found that a large portion of MDSCs was PD-L1 positive in hepatocellular carcinoma^19^.

The immune suppressive function of PD-L1 on MDSCs varied according to tumor type and cellular context. PD-L1 on MDSCs induced by the EL4 tumor cells did not exhibit suppressive activity against T cell activation in vitro under normoxia culture conditions^20^, while PD-L1^+^ MDSCs from AT3 tumor cell-conditioned medium were more immune suppressive than PD-L1^-^ MDSCs in vitro^21^. In this study, we investigated the suppressive function of PD-L1 on MDSC induced by HeLa cells, and showed for the first time that blocking PD-L1 expression with a neutralizing mAb only mildly decreased MDSC-mediated suppressive activity in vitro, suggesting that the suppressive function of PD-L1 on HeLa-induced MDSCs was very limited. The findings partly explained the low response rate of anti-PD-L1/PD-1 immunotherapy in the MDSCs rich tumor environment of cervical cancer.

ATRA is one of the few clinically available drugs that could modulate myeloid cells in both animal models and human patients^8^. It mediates potent myeloid cell differentiating effects in the setting of acute promyelocytic leukemia and is well tolerated with other immunotherapy, suggesting that it could be integrated into immunotherapeutic regimens for cancer^22^. However, no experiments have been designed to test the possibility of using ATRA to increase the response rate of PD-1/PD-L1 inhibitors in cervical cancer.

There are multiple immunosuppressive mechanisms that can be used by MDSCs. l-Arginine depletion by Arg1 and iNOS as well as ROS upregulation through NOX2, are two of the most important pathways to be reported in MDSCs^23^. In our research, ATRA decreased the expression of genes associated with immune suppression in CD33^+^ myeloid cells including Arg-1, iNOS, ROS and PD-L1. The low expression of multiple suppressive molecules supported direct depletion of MDSC, rather than suppression of specific gene expression in MDSCs. The full mechanism that underlies this effect remains to be elucidated. One putative mechanism is that ATRA mediates the differentiation of MDSC into mature myeloid cells through activation of ERK1/2 MAPK^24^. Experiments with adoptive transfer demonstrated that ATRA differentiated MDSCs into mature DC, macrophages, and granulocytes^25^. In our experiment, increased expression of HLA-DR after ATRA treatment in vitro, also supported a trend for increased DCs levels.

PD-L1 expression in tumor cells is believed the prerequisite for PD-L1/PD-1 immunotherapy in cancer^26^. In Keynote 158 (NCT02628067), 98 recurrent or metastatic cervical cancer patients were enrolled. With a median follow-up time of 11.7 months, the overall response rate was 14.3%, whereas no response was observed in patients without PD-L1 expression in tumor cells^27^. Our data showed that although ATRA significantly reduced the PD-L1 expression in MDSCs, it had no influence on PD-L1 expression in cell line HeLa in vitro. All these in vitro data collectively suggested that ATRA treatment abolished the immunosuppressive action of MDSC and could potentially be employed as an immunotherapeutic regimen.

In our experiment, although ATRA single agent blocked MDSCs immunosuppressive functions and increased T cells frequency, it still could not control tumor growth significantly. A crucial mechanism may be the negative regulation of effector T-cell response via immune checkpoints. PD-1/PD-L1 act as negative regulators of T-cell function and have been associated with immune evasion in cancer. Interestingly, the PD-L1 expression level increased somewhat after ATRA treatment in murine tumor models. Because ATRA had no effect on PD-L1 expression in Hela cells in vitro, the increased PD-L1 expression in tumors could be a form of adaptive immune resistance to the enhanced T cell activation.

Cancers have been categorized into four different tumor microenvironments based on the presence of TILs and PD-L1 expression, and of these, Type I tumors (PD-L1^+^, TILs^+^) are the most likely to benefit from anti-PD-1/L1 blockade, as they are “warm tumors” with increasing intratumor T cells^28,29^. Here, the lymphocyte predominant microenvironment and PD-L1 expression after ATRA treatment, provided a rationale for combining ATRA and anti-PD-1/L1 blockade.

CD62L is a marker found on naive T cells and CD62L expression is lost following T cell activation. A recent study found that in patients with non–small lung cancer, nivolumab responders presented significantly higher CD62L^low^ cell percentages in the populations of CD4^+^ T cells and CD8^+^ T cells^30^. Similarly, in our experiment we found higher proportion of CD62L negative cells in both CD4^+^ T cells and CD8^+^ T cells after combination treatment. However, the effect of CD62L expression on cancer immunotherapy was still controversy and Nakajima et al. showed that high CD62 expression on T cells improved the efficacy of cancer immunotherapy in mouse models^31^. These discordant findings showed CD62L expression might not be the only factor for the efficiency of cancer immunotherapy. CD107a, the marker of cytotoxic degranulation was also significantly upregulated on CD8^+^ T cells in combination treatment group. After combination treatment, CD62L shedding, increased expression of CD107a in T cells, as well as the elevated IFN-γ and TNF-α level partly account for the significant tumor growth inhibition. For checkpoint inhibitors rely primarily on pre-existing TILs, we suppose that the effective anti-PD-L1 treatment here may be due to the eliminating of immune-suppressive MDSCs.

Some limitations in our research need to be addressed. First, only one tumor cell line, HeLa, was used in the in vitro experiments. Second, the induced human MDSCs may have some biological differences from MDSCs present in tumor microenvironment. Thirdly, in our research, the mouse U14 cell line was HPV negative. Because HPV on its own can drive PD-L1 expression, there must be some differences in PD-L1 expression levels between the mouse model and human cervical cancer^32^.

In summary, our study provided the first pre-clinical evidence that treatment with ATRA could overcome the low anti-PD-L1 antibody response in cervical cancer treatment.

## Supplementary Information

Supplementary Information.

## Data Availability

The datasets used and/or analysed during the current study available from the corresponding author on reasonable request.
