# Supplementary material for: Inhibition of myeloid-derived suppressive cell function with all-trans retinoic acid enhanced anti-PD-L1 efficacy in cervical cancer
Source: Sci Rep. 2022 Jun 10;12:9619. doi: 10.1038/s41598-022-13855-1 (PMC9187659; doi:10.1038/s41598-022-13855-1)
Supplement: Supplementary file 1 — Supplementary Information. [file 41598_2022_13855_MOESM1_ESM.docx]

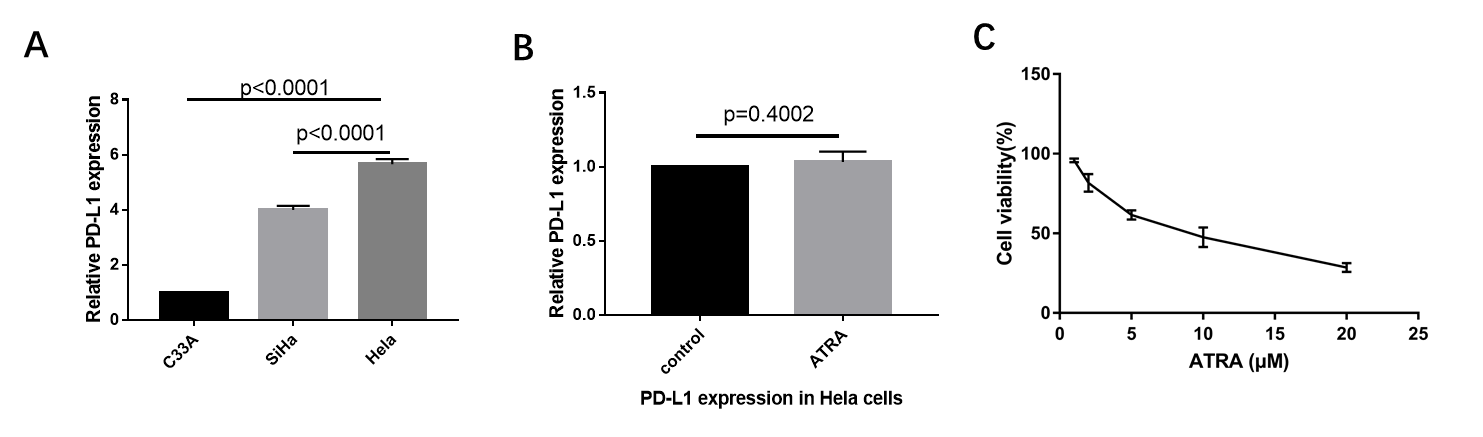


Supplementary Fig 1. ATRA had no influence in the PD-L1 expression on Hela in vitro. (A) qRT-PCR analyzed PD-L1 mRNA expression in HPV-positive and HPV-negative cervical cancer cell lines. (B) PD-L1 expression level had no change by qRT-PCR when treated with 2uM ATRA. (C) The cell proliferation inhibition rate of Hela cells was analyzed when treated with different concentrations of ATRA. (Data are representative from 4 separate experiments).


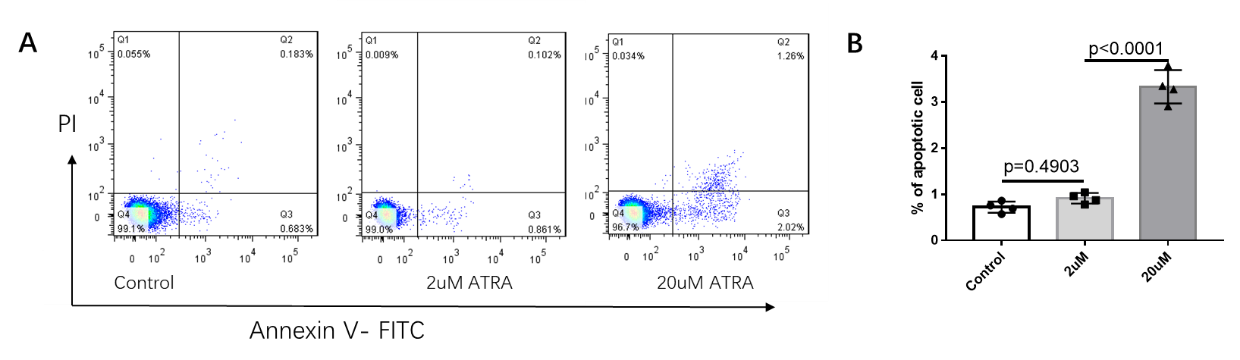


Supplementary Fig 2. ATRA had no influence in the apoptosis of Hela cells in vitro. (A) The representative flow cytometry dot plots of apoptosis were shown when HeLa cells treated with different concentration of ATRA. (B) The bar chart showed the apoptosis of Hela in different concentration of ATRA. Data are representative from 4 separate experiments. Data are shown as mean ± SD.


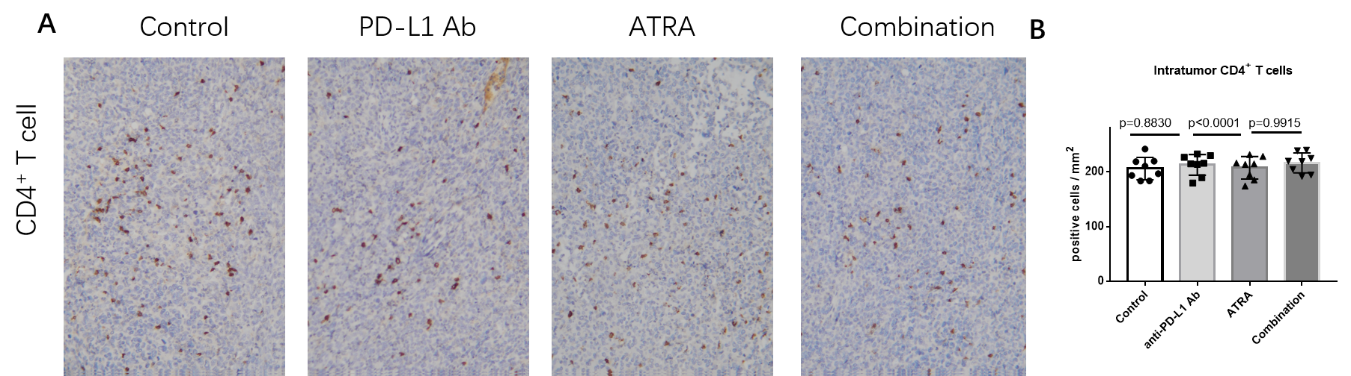


Supplementary Fig 3. Change in CD4^+^ T cells in tumor beds after treatment. Representative immunohistochemical images of CD4^+^ T cells in the tumor beds from different groups (A) and the comparison of CD4^+^ T cells numbers in different groups (B). Data are shown as mean ± SD (8 mice/group).


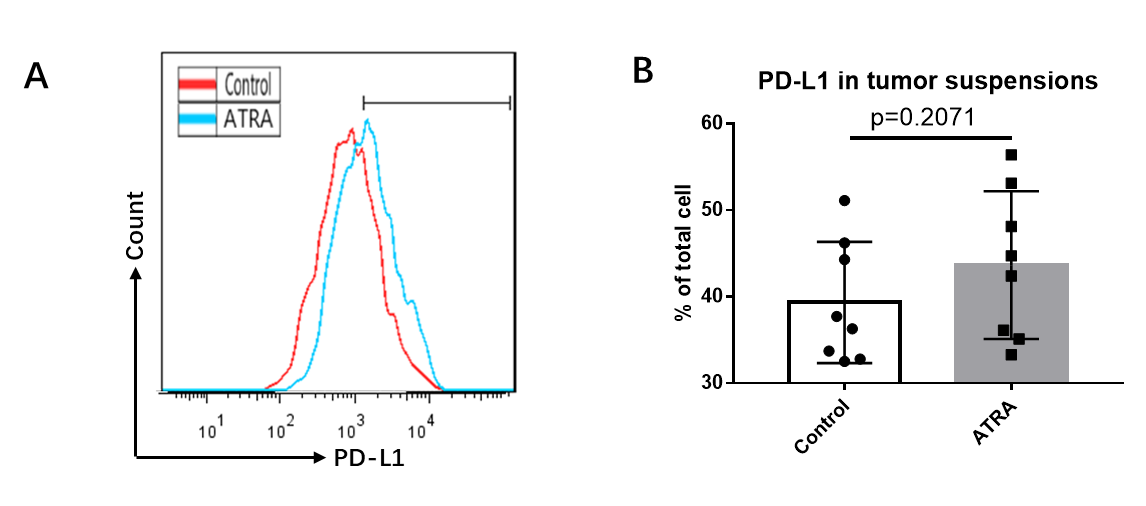


Supplementary Fig 4. The change of PD-L1 expression level was not significant after ATRA treatment in murine tumor models. Representative histograms of PD-L1 expression (left curve graph) and quantification (right panels). PD-L1 expression in tumor suspensions was quantified with or without ATRA. Data are shown as mean ± SD (8 mice/group, 2 repetitions with similar results).

Supplemental Table 1. Patient characteristics

| Characteristics of patients | Number/Value |
| --- | --- |
| Age(years) |  |
| <40 | 2 |
| ≥40 | 17 |
| Figo stage |  |
| IB2- IIA | 8 |
| IIB- III | 11 |
| Histological stage |  |
| I- II | 13 |
| III | 6 |

Supplementary Table 2: Human Primers (5’ **🡪** 3’)

| L-arginase1 Forward | GTT TTG ATG TTG ACG GAC TG |
| --- | --- |
| L-arginase1 Reverse | GTT CAC TGT TCG AGT TAC TT |
| iNOS Forward | CGG TGC TGT ATT TCC TTA CGA GGC GAA GAA GG |
| iNOS Reverse | GGT GCT GCT TGT TAG GAG GTC AAG TAA AGG GC |
| NOX2 Forward | TGC TAG AGA AAA AAT CAA GAA |
| NOX2 Reverse | CGC CAA AAC CGA ACC AAC CTC |
| PD-L1 Forward | GCG AAT TAC TGT GAA AGT CAA TGCC |
| PD-L1 Reverse | TGG TCA CAT TGA AAA GCT TCT CCTC |
| GAPDH Forward | GTC TCC TCT GAC TTC AAC AGC G |
| GAPDH Reverse | ACC ACC CTG TTG CTG TAG CCA A |
